# Genome-Wide Identification, Classification, and Expression Analysis of the *Hsf* Gene Family in Carnation (*Dianthus caryophyllus*)

**DOI:** 10.3390/ijms20205233

**Published:** 2019-10-22

**Authors:** Wei Li, Xue-Li Wan, Jia-Yu Yu, Kui-Ling Wang, Jin Zhang

**Affiliations:** 1 College of Landscape Architecture and Forestry, Qingdao Agricultural University, Qingdao 266000, China; lwcsu_caf@163.com (W.L.); wanxueli86@163.com (X.-L.W.); yjy13583204761@163.com (J.-Y.Y.); 2 State Key Laboratory of Tree Genetics and Breeding, Key Laboratory of Tree Breeding and Cultivation of the National Forestry and Grassland Administration, Research Institute of Forestry, Chinese Academy of Forestry, Beijing 100091, China; 3 Biosciences Division, Oak Ridge National Laboratory, Oak Ridge, TN 37831, USA

**Keywords:** heat shock factor, *Dianthus caryophyllus*, abiotic stresses, gene expression

## Abstract

Heat shock transcription factors (Hsfs) are a class of important transcription factors (TFs) which play crucial roles in the protection of plants from damages caused by various abiotic stresses. The present study aimed to characterize the *Hsf* genes in carnation (*Dianthus caryophyllus*), which is one of the four largest cut flowers worldwide. In this study, a total of 17 non-redundant *Hsf* genes were identified from the *D. caryophyllus* genome. Specifically, the gene structure and motifs of each *DcaHsf* were comprehensively analyzed. Phylogenetic analysis of the *DcaHsf* family distinctly separated nine class A, seven class B, and one class C *Hsf* genes. Additionally, promoter analysis indicated that the *DcaHsf* promoters included various *cis*-acting elements that were related to stress, hormones, as well as development processes. In addition, *cis*-elements, such as STRE, MYB, and ABRE binding sites, were identified in the promoters of most *DcaHsf* genes. According to qRT-PCR data, the expression of *DcaHsfs* varied in eight tissues and six flowering stages and among different *DcaHsfs*, even in the same class. Moreover, *DcaHsf-A1, A2a, A9a, B2a, B3a* revealed their putative involvement in the early flowering stages. The time-course expression profile of *DcaHsf* during stress responses illustrated that all the *DcaHsfs* were heat- and drought-responsive, and almost all *DcaHsfs* were down-regulated by cold, salt, and abscisic acid (ABA) stress. Meanwhile, *DcaHsf-A3*, *A7, A9a, A9b*, *B3a* were primarily up-regulated at an early stage in response to salicylic acid (SA). This study provides an overview of the *Hsf* gene family in *D. caryophyllus* and a basis for the breeding of stress-resistant carnation.

## 1. Introduction

Plant growth and production are affected by abiotic stresses such as heat, cold, drought, and salinity [1,2,3]. Unlike animals, plants are sessile organisms. Consequently, to cope with environmental stresses, plants have evolved a series of defense or signaling mechanisms. Furthermore, each process involves different types of transcription factors (TFs). These include heat shock transcription factors (Hsfs), such as WRKY, MYB, AP2/ERF, and NAC, which regulate the expression of thousands of genes under various stress conditions [4,5,6]. In plants, the Hsf family is one of the most important TF families in plants involved in resistance to heat [7] and other abiotic stresses or chemical stressors, such as abscisic acid (ABA) and salicylic acid (SA) [8,9]. Hsfs regulate the expression of *Heat shock proteins* (*Hsps*) as well as other stress-responsive proteins, such as reactive oxygen species (ROS)-scavenging enzymes [ascorbate peroxidase (APX) and catalase (CAT)] [4]. Besides their roles in stress responses, Hsfs are also involved in plant growth and development [10,11,12].

Similar to many other TFs, *Hsfs* are a part of an evolutionarily conserved gene family. *Hsf* genes are composed of several structurally and functionally conserved domains, including DNA-binding domains (DBD), N-terminal adjacent bipartite oligomerization domains (HR-A/B), nuclear localization signals (NLS), nuclear export signals (NES), C-terminal activator peptide proteins (AHA), and repressor domains (RD) [13]. Among these conserved domains, DBD is characterized by a central helix–turn–helix motif and is responsible for binding to the heat shock elements (HSEs) of the target genes [7]. Notably, the HSEs are palindromic binding motifs (5’-AGAAnnTTCT-3’) conserved in the promoters of heat stress-inducible genes [7,14]. According to the flexible linker of variable lengths (about 15–80 amino acids) and HR-A/B regions, plant *Hsfs* can be divided into at least three types, i.e., class A (subclasses A1, A2, A3, A4, A5, A6, A7, A8, and A9), class B (subclasses B1, B2, B3, and B4), and class C (subclasses C1 and C2) [15,16,17].

The size of the *Hsf* gene family varies significantly in different plant species. For instance, there are 22 *Hsf* members in the model plant *Arabidopsis thaliana* [18], 25 members in *Oryza sativa* [18], 16 members in *Medicago truncatula* [19], 26 members in *Glycine max* [20], 25 members in *Zea mays* [21], 25 members in *Malus domestica* [22], 21 members in *Cucumis sativus* [23], 28 members in *Populus trichocarpa* [24], 40 members in *Gossypium hirsutum* [25], and 56 members in members in *Triticum aestivum* [26]. To date, the largest *Hsf* gene family has been identified in *Brassica napus*, with 64 *Hsf*s [27].

Carnation (*Dianthus caryophyllus* L.) is a major floricultural crop and one of the four largest cut flowers [28,29]. Until now, more than 300 *Dianthus* species have been identified worldwide. Carnations are cultivated widely for their attractive characteristics such as flower color, flower size, fragrance, and flower longevity. However, the vegetative and reproductive growth of carnations are severely impaired in heat stress conditions, resulting in flower wilting and quality decline [30]. The completion of the draft genome sequence of *D. caryophyllus* L. has greatly facilitated the identification of *Hsfs* at the whole-genome level and it is extremely important to study the heat-resistant mechanism of carnation [31]. To our knowledge, there are no reports on the identification and functional analysis of carnation *Hsfs* to date. In this study, we aimed to comprehensively study the structural and expression profiles of the *Hsf* gene family in *D. caryophyllus*. A total of 17 putative genes were identified and characterized as members of the *Hsf* gene family from *D. caryophyllus*. Additionally, we performed bioinformatic analyses of phylogenetic relationships, conserved domains, motifs, and other. Furthermore, the expression level of these genes in various tissues and in response to abiotic stresses were compared. Our results will be a reference and provide valuable information for the functional analysis of the *Hsf* genes in *D. caryophyllus*.

## 2. Results 

### 2.1. Identification of DcaHsfs in Carnation

The amino acid sequences of putative Hsf proteins were examined using the conserved Hsf domain (PF00447) from the carnation database (DB, http://carnation.kazusa.or.jp). Additionally, searches using the BLASTP program resulted in the identification of putative *Hsf* gene candidates. In total, 17 proteins were retrieved as DcaHsfs in *D. caryophyllus*.

**Figure 1:**
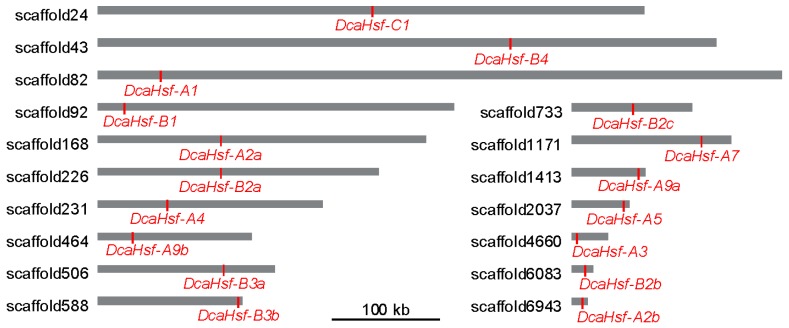
Scaffold locations of *DcaHsfs*. Bars represent the scaffolds, *DcaHsfs* are marked by redlines.

The physical and chemical properties of the 17 DcaHsfs were analyzed (Table 1). The DcaHsfs ranged from 133 amino acids (aa; DcaHsf-C1, incomplete) to 495 aa (DcaHsf-A5) in length. The predicted isoelectric points (pI) varied from 4.74 (DcaHsf-A1) to 8.88 (DcaHsf-B3a and DcaHsf-B3b), and the molecular weight (MW) varied from 15.89 kDa (DcaHsf-C1) to 54.52 kDa (DcaHsf-A5). The instability index, i.e., the stability of the protein in a test tube, indicated that all DcaHsfs were unstable, except for DcaHsf-A9b and DcaHsf-B1. The GRAVY value reflects the hydropathicity of a protein; the low GRAVY values (<0) of DcaHsfs suggest that all DcaHsfs are hydrophilic. The total number of negatively charged residues (Asp + Glu, n.c.r.) and the total number of positively charged residues (p.c.r.) of class A were all greater than those of class B and C. These differences might be caused by differences in the amino acid composition of the non-conserved region. We determined the scaffold locations of *DcaHsfs* on the basis of the information from the Carnation genomic database. We mapped 17 *DcaHsfs* to 17 scaffolds, and these genes were distributed evenly in the Carnation genome (Figure 1).

### 2.2. Phylogenetic and Sequence Conservation Analysis of DcaHsfs

**Figure 2:**
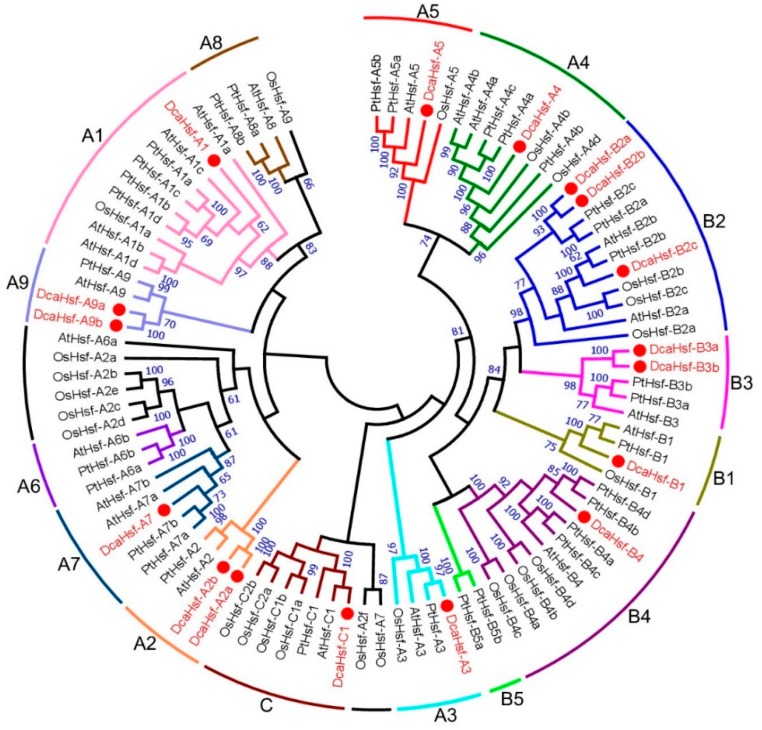
The phylogenetic tree of Hsf proteins. The phylogenetic tree of Hsf proteins in carnation and other plant species was generated by MEGA 7 using the neighbor-joining method. Dca, *D. caryophyllus*; At, *Arabidopsis thaliana*; Os, *Oryza sativa* and Pt, *Populus trichocarpa*.

To explore the phylogenetic relationship of *Hsfs* in *D. caryophyllus* and other species, the amino acid sequences of Hsfs from *A. thaliana*, *O. sativa*, and *P. trichocarpa* were used, together with those of DcaHsfs, as a means to construct a phylogenetic tree. In this study, 21 Hsf proteins from *A. thaliana* [18], 25 from *O. sativa* [18], 31 from *P. trichocarpa* [32], and 17 from *D. caryophyllus* were utilized for the phylogenetic analysis. A total of 94 Hsf proteins from the four species were clearly divided into three classes (class A, B, and C) with well-supported bootstrap values (Figure 2).

In *D. caryophyllus*, 9 DcaHsfs out of 17 proteins belonged to class A, making it the largest subclass, followed by 7 DcaHsfs belonging to class B. The number of class B Hsfs in *D. caryophyllus* (7) was greater than that in *Arabidopsis* (5). The class C Hsf was present as a single copy in *D. caryophyllus*, *Arabidopsis*, and *P. trichocarpa*, whereas four copies of class C Hsfs were discovered in *O. sativa*. However, none of the DcaHsfs belonged to subclasses A6 and A8. Sequence conservation among DcaHsfs was also supported by their identity at the amino acid level. Detailed information on the identity of AtHsfs, OsHsfs, and PtHsfs amino acid sequences is illustrated in Table S1.

### 2.3. Structural and Motif Analysis of DcaHsfs

**Figure 3:**
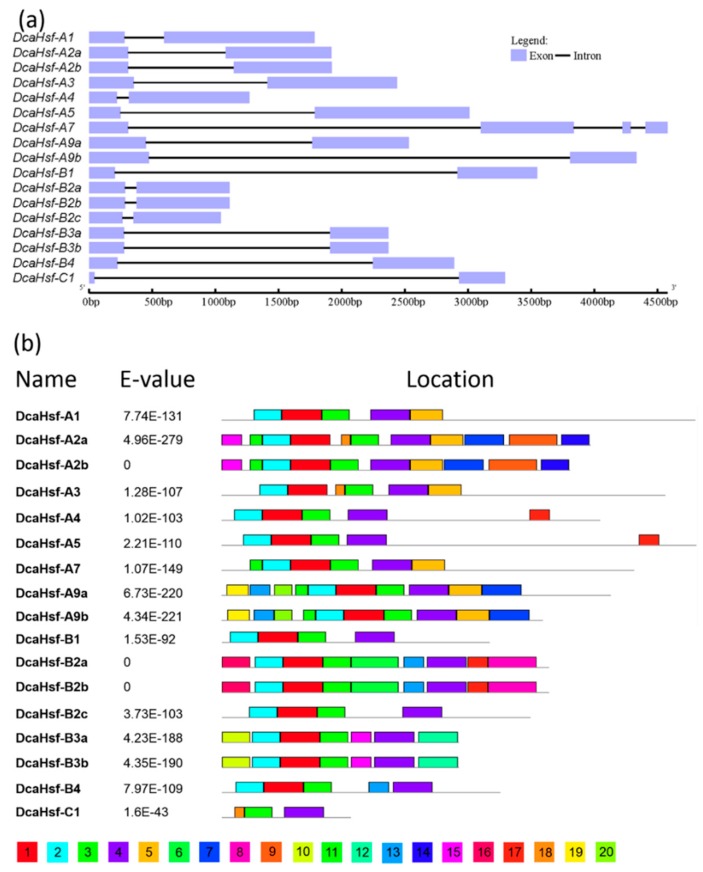
Intron and exon structure (**a**) and amino acid motifs (**b**) of members of the DcaHsf family. (**a**) Boxes and lines represent exons and introns, respectively. (**b**) A total of 20 conserved motifs were identified using Multiple Em for Motif Elicitation (MEME).

The structural diversity of the DcaHsf family was analyzed in terms of the exon/intron arrangement of the coding sequences. The number of introns in *DcaHsfs* ranged from one to three. The detailed gene structure of *DcaHsfs* is pictured in Figure 3a. Three introns were identified in *DcaHsfs-A7*, whereas all the other *DcaHsfs* had only one intron. Most closely related *DcaHsfs* in the same class or subfamily shared a similar gene structure in terms of intron number and intron and exon length (Figure 3a).

To investigate the protein sequence features of DcaHsfs, 20 different motifs were identified in DcaHsfs, with lengths ranging from 10 to 50 aa. (Figure 3b, Table 2). All members showed similar motif composition, but small differences between different groups were also found (Figure 3b). The conserved motifs in *Hsf* genes indicated that all DcaHsfs contained motif 1, motif 2, motif 3, and motif 4, except for DcaHsf-C1. Additionally, some motifs were only discovered in a certain subfamily of DcaHsfs. For instance, motif 6, motif 8, motif 9, and motif 14 were present in the B2 subfamily, whereas motif 10 and motif 12 were present in the B3 subfamily. Specifically, the phylogenetic analysis showed that the same clusters of DcaHsfs shared similar conserved domain composition. This indicates that *Hsf* genes are evolutionarily well conserved or possess similar regulatory functions in *D. caryophyllus* (Figure 3b). Additionally, motifs 1–3 represent the Hsf DBD domains (~100 aa). The DBD domain contains three α-helices and a four-stranded antiparallel β-sheet (α1-β1-β2-α2-α3-β3-β4) (Figure S1).

The 20 motifs consist of six different domains, including DBD, HR-A/B, NLS, NES, RD, and AHA domains. Among these, the highly structured DBD domain is the most conserved section in the DcaHsf family (Table 3). In addition to DBD, HR-A/B is critical for Hsf–Hsf interactions in the formation of a trimer [7], HR-A/B is also present in all DcaHsfs, and class A Hsfs have longer HR-A/B regions compared with class B and class C Hsfs (Figure S1, Table 3) Meanwhile, the other four conserved domains were only identified in specific DcaHsf members. The majority of class A DcaHsfs contained an NLS sequence rich in basic amino acid residues (K/R), except DcaHsf-A1/A3, whereas two or three NLS domains were located in seven DcaHsfs (A2a, A2b, A7, A9a, A9b, B3a, and B3b). NLS domains were not identified in DcaHsf-C1 and in some class B proteins (DcaHsf-B1, B2a, B2b, B2c). NES motifs were found in nine DcaHsfs. Also, five Class B Hsfs, except DcaHsf-B2a and DcaHsf-B2b, contained an RD in the C-terminus, characterized by the tetrapeptide LFGV. Transcription activator AHA motifs were located in class A DcaHsfs (Figure S1, Table 3). Sequence conservation among DcaHsfs was also supported by their identity at the amino acid level (0.023–0.83, Table S2). Four pairs of DcaHsfs (A2a–A2b, A9a–A9b, B2a–B2b, and B3a–B3b) exhibited high sequence identity (Table S2).

### 2.4. Cis-Acting Element Analysis in the Promoters of DcaHsfs

**Figure 4:**
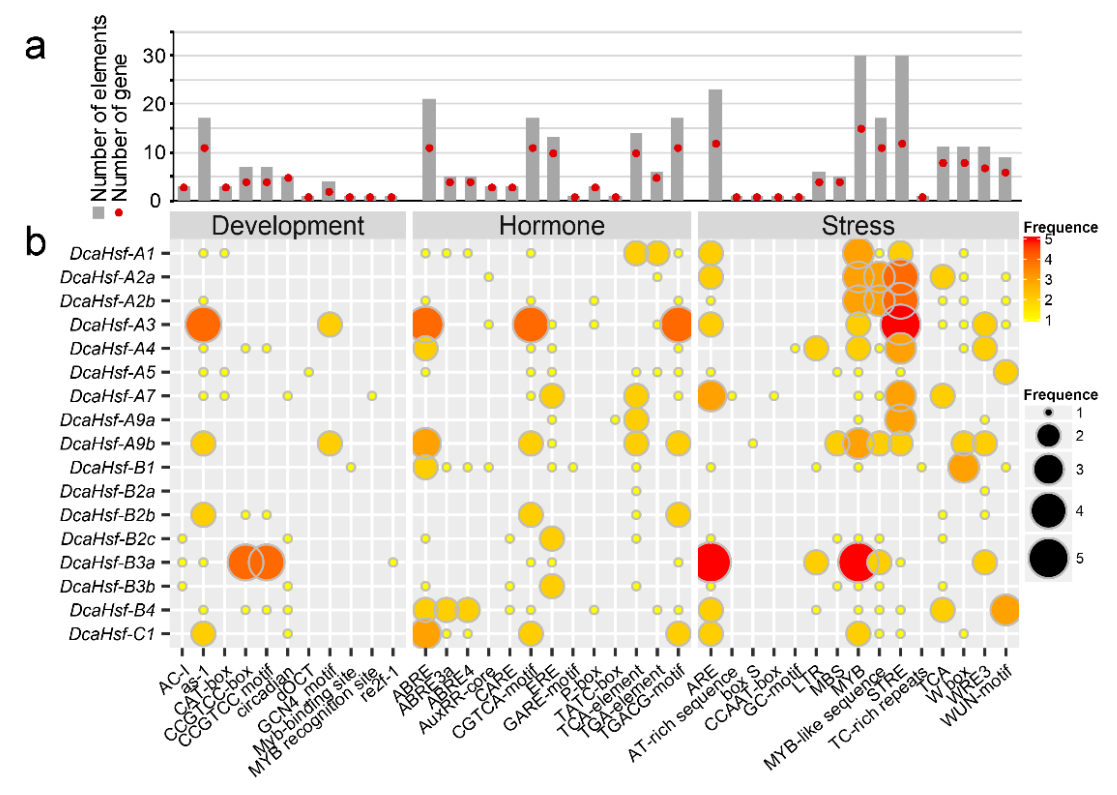
*Cis*-regulatory elements in the promoter region of *DcaHsfs*. (**a**) Number of each *cis*-acting element in the promoter region (1.5 kb upstream of the translation initiation site) of *DcaHsfs*. Statistics of the total number of *DcaHsfs* including the corresponding *cis*-acting elements (red dot) and the total number of *cis*-acting elements in the *DcaHsf* gene family (gray box). (**b**) Frequency of the *cis*-acting elements in each gene. Based on the functional annotation, the *cis*-acting elements were classified into three major classes: stress-, hormone-, and development-related *cis*-acting elements.

To predict the biological function of *DcaHsfs*, 1500-bp upstream sequences from the translation start sites of *DcaHsfs* were analyzed through the PlantCARE database. The promoter of each *DcaHsfs* consists of several *cis*-acting elements, such as phytohormone-, abiotic stress-, and developmental process-related elements. As illustrated in Figure 4, the MYB element, ARE element (essential for anaerobic induction), and STRE element (activated by heat shock, osmotic stress, low pH, and nutrient starvation) were identified in the promoters of 15, 12, and 12 *DcaHsf* genes, respectively. The promoters of 11 *DcaHsfs* contained the ABA-responsive element (ABRE), methyl jasmonate (MeJA)-responsive element (CGTCA-motif), and TGACG motif involved in MeJA responsiveness (Figure 4). Also, the ethylene-responsive element (ERE), *cis*-acting element involved in salicylic acid responsiveness (TCA-element), stress-inducible element (TCA), wounding and pathogen responsiveness elements (W-box) were all found in 10, 10, 8, and 8 *DcaHsfs*, respectively (Figure 4). In total, 17 *DcaHsf* promoters contained 30 MYB, 30 STRE, 23 ARE, 21 ABRE, and 17 CGTCA-motif elements (Figure 4). These findings demonstrate that *DcaHsfs* might be associated with various transcriptional regulations involving development, hormones, and stress responses.

### 2.5. The Expression Pattern of DcaHsfs in Different Tissues and Flower Development

**Figure 5:**
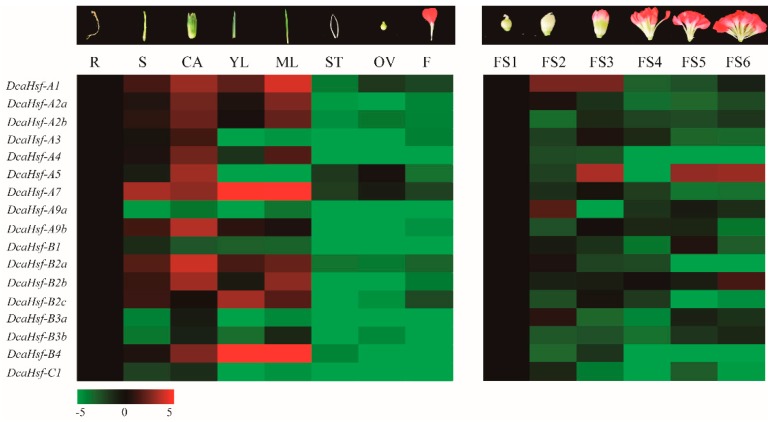
The expression levels of *DcaHsfs* in different tissues and flowering stages. The different colors correspond to log_2_-transformed fold change, green indicates down-regulation, and red represents up-regulation.

To elucidate the tissue-specific expression patterns of *DcaHsfs*, qRT-PCR was utilized to determine the expression levels of 17 *DcaHsfs* in 8 carnation tissues [root (R), stem (S), calyx (CA), young leaf (YL), mature leaf (ML), stigma (ST), ovary (OV), and flower (F)] and at 6 flowering stages (FS1, FS2, FS3, FS4, FS5, and FS6) (Figure 5, Table S4). Interestingly, the expression levels differed in different tissues and flowering stages, and the expression patterns of different members of DcaHsfs also differed, even for the same class. Among the different tissues, *DcaHsf-A1, A2a, 2b, A7, A9b, B2a, 2c*, *B4* were up-regulated in S, CA, YL, and ML. Meanwhile, all *DcaHsf*s were down-regulated in ST, OV, and F (Figure 5). Twelve *DcaHsfs* were more highly expressed in CA, and 10 out of 17 *DcaHsfs* had higher expression levels in ML (Figure 5). Some genes demonstrated tissue-specific expression patterns. For instance, *DcaHsf-A9a, B1, B3a, B3b, C1* were up-regulated in R, and *DcaHsf-A3, A4, A5* were expressed at high levels in CA.

During the six flowering stages of carnations, all *DcaHsfs* showed relatively high expression levels at FS1. Additionally, *DcaHsfA1, A2a, A9a, B2a, B3a* were up-regulated at FS2 (Figure 5), implying that these genes may be involved in the early development of carnation flowers. In contrast, *DcaHsf-A5* and *DcaHsf-B2b* exhibited a high expression level at FS6 (Figure 5).

### 2.6. DcaHsfs Response to Various Stresses

**Figure 6:**
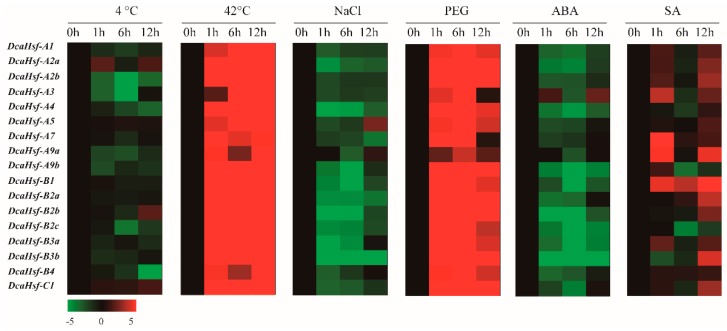
Expression levels of *DcaHsfs* under various abiotic stresses, determined by qRT-PCR. The different colors correspond to log_2_-transformed fold change, green indicates down-regulation, and red represents up-regulation.

To determine the potential roles of the *DcaHsfs* in plant responses to various environmental stresses, qRT-PCR was conducted on the 17 *DcaHsfs* using the leaves of carnations exposed to heat, cold, drought, salt, ABA, and SA. The results illustrated that almost all *DcaHsfs* revealed three types of expression patterns under different stress conditions: (1) the expression of all genes was up-regulated; (2) the expression of all genes was down-regulated; and (3) some genes were expressed at higher levels in the early stage of stress, while others were up-regulated in the later stage of stress (Figure 6). Regarding the first category (1), all *DcaHsfs* were up-regulated after leaf exposure to heat and polyethylene glycol (PEG) treatments (Figure 6). For the second category of genes (2), almost all *DcaHsfs* displayed a decrease in their expression levels under cold, salt, or ABA stresses (except for individual *DcaHsfs*) (Figure 6). For example, four *DcaHsfs* (*DcaHsf-A2a*, *A5*, *B2b*, *C1*) were slightly induced at different time points at 4 °C., while the transcription levels of the remaining 13 genes were down-regulated at the tested time points (Figure 6). *DcaHsf-A5* demonstrated higher transcript accumulation compared to the other genes at 12 h under 200 mM NaCl treatment. Meanwhile, *DcaHsf-A3* was slightly up-regulated at 12 h under ABA treatment (Figure 6). Finally, the genes in category (3) and the expression of *DcaHsf-A3*, *A7, A9a, A9b*, *B3a* were primarily up-regulated at the earlier stage of SA treatment, whereas other *DcaHsfs* were strongly up-regulated after 12 h of SA treatment (Figure 6). These findings indicate that *DcaHsf* genes might play crucial roles in different stress response pathways.

## 3. Discussion 

### 3.1. Characterization of the Carnation Hsf Genes Family

Hsfs exist extensively in all plant species and act as the key regulatory components involved in various abiotic stresses to protect the plant cellular machinery under stress conditions [4,13,26]. In this study, a comprehensive genome-wide analysis of the *DcaHsf* family in carnations was carried out for the first time. A total of 17 *DcaHsf* genes were identified from the Carnation genome database [31]. The size of the carnation *Hsf* gene family is smaller compared with that of three other plant species, i.e., *A. thaliana*, *O. sativa*, and *P. trichocarpa*. Meanwhile, all four species have a similar subfamily distribution, which indicates that parallel evolutionary events of *Hsf* genes occurred in dicots and monocots. Additionally, the subclasses A6 and A8 are absent in carnation, and the diversification of *Hsf* members could provide some clues about the biological function of the corresponding *Hsf* counterparts in carnation. This suggests that gene loss and gene duplication events occurred at different stages of the evolutionary process, resulting in *Hsf* diversity [18] (Table S1).

### 3.2. Cis-Element Analysis in the Promoters of DcaHsfs

The number and form of *cis*-elements in promoter regions might play an essential function in the regulation of gene expression related to metabolic pathways [33]. The results illustrate that abiotic stress-related *cis*-elements, including MYB, STRE, ARE, ABRE, CGTCA-motif element, ERE, TCA-element, and W-box, are major regulatory elements in *DcaHsfs* promoters activated by heat shock or other abiotic stress. The presence of these stress-related elements is related to the expression response of *DcaHsf*s to heat, drought, ABA, and SA treatments. STRE is a marker element for plant Hsf proteins, which has been located in the promoters of the 17 *DcaHsf*s (Figure 4). In our study, a large number of STRE elements were identified in the promoter of 12 *DcaHsf* genes, which coincides with their expression (Figure 6). These findings suggest that STRE plays a vital role in transcriptional regulation under heat conditions in carnation. *DcaHsf* subclass A promoters contained MYB binding sites which participate in drought, low temperature, salt, ABA, and gibberellic acid (GA) stress responses [34]. We found that 15 *DcaHsfs* included 30 MYB binding sites in their promoter regions. However, the presence of MYB elements seems to be correlated with the positive regulation of *DcaHsfs* during drought and the negative regulation of *DcaHsfs* in response to salt and ABA treatments (Figure 6, Table S3). Other abiotic stress-related *cis*-elements, including the CGTCA-motif, TGACG-motif, ERE element, and TCA-element, were also major regulatory elements identified in *DcaHsfs*. Furthermore, the presence of these stress-related elements appears to be correlated with MeJA, SA, and stress responsiveness, suggesting their potential roles in the response to pathogen infections. Consequently, *DcaHsfs* could be taken as candidate genes to understand the responses to drought and other biotic stresses.

### 3.3. Structural Analysis of DcaHsfs

The detailed knowledge of *A. thaliana*, *O. sativa*, and *P. trichocarpa Hsf* functional domains enabled us to analyze similar domains in *D. caryophyllus Hsf* gene family. It has been reported that the number of introns both regulate gene expression and participate in gene evolution [35]. Analysis of *Hsf* gene structure revealed that 16 of 17 *DcaHsfs* have one intron in their DBD domain (Figure 3a), which is an evolutionarily conserved intron [36]. However, *DcaHsf-A7* contains three introns (Figure 3a), which might affect its expression under stress conditions. All 17 DcaHsfs proteins contain the necessary DBD domain and specific protein domains (HR-A/B, NLS, NES, RD, and AHA) (Table 3, Figure 3b), which provide the structural basis for their conserved function [22]. The Hsf DBD domain of approximately 100 amino acid residues is highly conserved in different organisms, from plants to animals [7]. However, the DBD of DcaHsf-C1 contains only 44 aa and is shorter than the other DcaHsfs, lacking the full α1-helix, β1-sheet, β1-sheet, and α2-helix. Notably, this might be caused by the current genome assembly. It is interesting that AHA, an essential domain for the activator function in the HsfA class [7], was not found in several members of class A DcaHsfs (A1, A3, A9a, and A9b) (Table 3). The members of Hsfs lacking AHA domains might contribute differently to the activator function or bind to other HsfAs to form hetero-oligomers [18].

### 3.4. DcaHsfs Involvement in Carnation Development Processes 

The expression patterns of *DcaHsfs* in seven different organs or tissues uncovered that *DcaHsfs* have different expression profiles in carnation. This suggests that they may participate in various developmental processes or regulatory pathways. In this study, nearly all the *DcaHsfs* were found to display high transcription levels in ML and at FS1 (Figure 5). Within the potato *HsfA1* group, *StHsf002* is highly expressed in flowers, petals, and sepals, whereas *StHsf003* is highly expressed in roots, flowers, carpels, and sepals [37]. In our study, *DcaHsf-A1* demonstrated up-regulation in S, CA, YL, and ML (Figure 5). *Phyllostachys edulis PheHsfA2a-2* is predicted to play an important role in flower and shoot development [38] and *Cicer arietinum CarHsfA2* is up-regulated in shoot, root, and flower [12]. Their orthologs in carnations, *DcaHsf-A2a, A2b*, were constitutively expressed in S, CA, YL, and ML at relatively high levels (Figure 5). These findings indicate that the members of the *Hsf-A1* and *A2* sub-families are conserved and involved in the development of vegetative organs. 

*HsfA5* has been reported to play a vital role in stress tolerance during anther/pollen development as well as in other stages of plant reproduction in tomato and *Arabidopsis* [22,39]. In this study, *DcaHsf-A5* was highly expressed in CA and OV (Figure 5). This implies that the function of *DcaHsf-A5* might be conserved for regulating reproductive organ development and the growth of carnation. *Salix suchowensis SsuHsf-A9* is specifically expressed in the female catkin [32]. In *Populus* female catkin development, *PtHsf-A9* displays relatively high transcription levels [40]. Our results indicate that carnation *DcaHsf-A9b* was up-regulated in S and CA and is possibly widely involved in the development of both vegetative and reproductive tissues. 

For Class B *Hsfs*, Chickpea *CarHsfB2c* is highly expressed in the late flowering stages, while *CarHsfB2a* is expressed in root, flower, pod wall, and grain. *CarHsfB4b* is specifically expressed in flower and grain [12]. In carnations, *DcaHsfs-B2a, B2c, B4* are highly expressed in S, CA, YL, and ML. However, *DcaHsf-B2a/B3a* and *DcaHsf B2b* are highly expressed in FS2 and FS6, respectively (Figure 5). This indicates that members of the Class B *DcaHsfs* might be widely involved in the development of both vegetative and reproductive organs and tissues. The expression patterns of *Hsf-C1* genes were diverse in different tissues. For example, the transcripts of *Vitis pseudoreticulata VpHsfC1a* remain at relatively lower levels (even undetectable) in roots, stems, leaves, and tendrils [41]. Similarly, *SaHsfC1a* is expressed at low levels in all the tested tissues in *Sedum alfredii* [42]. Carnation *DcaHsf-C1* was down-regulated in almost all tested tissues (except for R) (Figure 5), which is consistent with the expression pattern of *SaHsfC1b* [42]. Specifically, it may be attributed to the fact that *DcaHsf-C1* acts as a negative regulator in the development of organs. 

### 3.5. DcaHsfs are Involved in Carnation Stress Response 

The genome-wide expression profile analyses indicated that the majority of the *Hsf* genes are involved in heat, cold, drought, and salt stress responses [22,26]. Under heat or other stress conditions, plant *Hsfs* display diversity in patterns of expression [14]. In our study, all 17 *DcaHsfs* were found to be induced by a high temperature of 42 °C (Figure 6), which is in agreement with a previous study [43]. All *DcaHsfs* accumulated during drought treatment (Figure 6), and a previous study revealed that approximately 90% of sesame *Hsfs* are drought-responsive [44]. *DcaHsf-A2a, A2b* revealed to be strongly induced under heat stress conditions. This indicates that HsfA2 is a dominant regulator during the heat stress response in carnation, which is consistent with the studies of *Arabidopsis*, tomato, apple, *Populus euphratica*, and *Phyllostachys edulis* [11,22,38,39,45]. *HsfA3* has been identified as an important player in the responses to heat, high salinity, and drought stresses in *Solanum lycopersicum* [46], whereas a similar function for *HsfA3* is not detected in tomato [14]. In this study, *DcaHsfA3* was also up-regulated in response to four analyzed abiotic stresses (heat, drought, ABA, and SA) (Figure 6). Group A4 Hsfs are involved in controlling reactive oxygen species homeostasis in plants, and group A5 Hsfs act as specific repressors of HsfA4 [47,48]. *Fragaria vesca FvHsfA4a*, *A5a* were both distinctly up-regulated in response to abiotic stresses such as cold, drought, and salt and hormone treatments (ABA, Eth, MeJA, and SA) [49]. Our data are highly similar, indicating *DcaHsf-A4* accumulation during heat, drought, and SA treatment, as well as *DcaHsf-A5* upregulation in response to cold, heat, drought, and salt and SA treatments (Figure 6). *Arabidopsis Hsf-A9a* is associated with ABA-mediated stress signaling and drought resistance [50]. Similarly, *DcaHsfA9a* was also induced in response to salt, ABA, and SA (Figure 6). Compared to Class A *Hsfs*, the members in Class B and C still have not been well studied. *Arabidopsis AtHsfB1a* and *F. vesca FvHsfB1a* were highly induced and accumulated in response to SA treatment [45,49,51]. Additionally, additional evidence demonstrated that *AtHsfB1a, B2b* are crucial components in primed defense gene activation and pathogen-induced acquired immune response [51]. Similarly, in this study, *DcaHsf-B1, B2a, B2b* accumulated at high levels at the later stage of SA treatment (Figure 6) Therefore, it is reasonable to speculate that *DcaHsf-B1, B2a, B2b* play a crucial role in the acquired immune response to pathogens. Additionally, *DcaHsf-C1* acts as a positive regulator of heat shock proteins under heat stress conditions or PEG stress. The expression of rice *OsHsfC1b* was induced by salt, mannitol, and ABA [52]. In *V. pseudoreticulata, VpHsfC1a* was up-regulated in response to ABA treatment but significantly down-regulated during both MeJA and Eth treatments [41]. However, the expression of *DcaHsf-C1* was not up-regulated by cold, ABA, or salt stress (Figure 6). We can speculate that *DcaHsf-C1* might be involved in ABA-independent pathways in carnations. However, gene expression is a complex biological process, and more thorough studies are required to decipher the regulatory mechanisms.

## 4. Materials and Methods 

### 4.1. Identification and Characterization of Hsf Genes in D. caryophyllus 

The protein and nucleotide sequences of *D. caryophyllus* were downloaded from the carnation DB (http://carnation.kazusa.or.jp). The conserved domain of Hsf DBD (Pfam: PF00447) was submitted as a query in a BLASTP search of the *D. caryophyllus* proteome. The SMART 7 software (http://smart.embl-heidelberg.de/) was used to identify integrated DBD domain and (HR-A/B) domain in the putative Hsfs. Candidate proteins without integrated DBD domain and HR-A/B domain were removed. The ExPaSy-Protparam tool (https://www.expasy.org/tools/ProtParam.html) was used to analyze the physical properties of the predicted Hsf proteins. 

### 4.2. Phylogenetic Analysis

Multiple sequence alignments of full-length Hsf proteins from *D. caryophyllus* and other three model species, i.e., *A. thaliana*, *O. sativa*, and *P. trichocarpa*, were performed using Clustal W2 (https://www.ebi.ac.uk/Tools/msa/clustalw2/, Dublin, Ireland). An unrooted neighbor-joining (NJ) phylogenetic tree was constructed using MEGA7.0 (Philadelphia, PA, U.S.A.) with 1000 bootstrap replicates. Distinctive names for each of the *Hsfs* identified in *D. caryophyllus* were given according to the classification of *Hsfs* in classes A, B, and C, referred to as *DcaHsf* genes.

### 4.3. Structural and Motif Analyses of DcaHsf Genes

The gene structures including exons and introns were displayed using Gene Structure Display Server (GSDS, http://gsds.cbi.pku.edu.cn/index.php, Beijing, China). The conserved motifs of DcaHsfs were defined by Multiple Em for Motif Elicitation (MEME, http://meme-suite.org/, U.S.A.) using the following parameters: number of repetitions = any, maximum number of motifs = 20, minimum width ≥10, maximum width ≤200, and only motifs with an *E*-value < 0.01 were retained for further analysis. NLS domains were predicted using cNLS Mapper software (http://nls-mapper.iab.keio.ac.jp/cgi-bin/NLS_Mapper_form.cgi, Tsuruoka, Japan). NES domains in the *DcaHsfs* were predicted with the NetNES 1.1 server software (http://www.cbs.dtudk/services/NetNES/, Lyngby Denmark).

### 4.4. Cis-acting Element Analysis of DcaHsfs

The 1500-bp sequence upstream from the initiation codon of each *DcaHsf* gene was obtained from the *D. caryophyllus* genome database. These sequences were used to identify *cis*-acting regulatory elements with the online program PlantCARE (http://bioinformatics.psb.ugent.be/webtools/plantcare/html/, Ghent, Belgium).

### 4.5. Plant Materials, Growth Conditions, and Stress Treatments

Tissue culture seedlings of carnation were grown in a chamber at Qingdao Agriculture University (Qingdao, China) under a 12 h light (300 μmol·m^−2^·s^−1^)/12 h dark cycle at 23–25 °C ambient temperature and 70% relative humidity. Various tissues, including the root (R), shoot (S), calyx (CA), young leaf (YL), mature leaf (ML), stigma (ST), ovary (OV), and flower petals (F), and six flowering stages (FS1, FS2, FS3, FS4, FS5, and FS6) were collected from the carnation seedlings. For abiotic stress and hormone treatments, the seedlings were treated at 42 °C (for heat stress), with 20% (*w/v*) polyethylene glycol (PEG) 6000 (for drought stress), 200 mM NaCl (for salt stress), 100 μM ABA, or 100 μM SA. The first or second tender leaves of the seedlings were collected at 0, 1, 6, and 12 h, immediately frozen in liquid nitrogen, and then stored at −80 °C for further analysis. Three biological replicates were performed for each sample.

### 4.6. RNA Isolation and Expression Analysis of DcaHsf Genes

Total RNA from carnation leaves was extracted using the Plant RNA Kit (Omega, Norcross, GA, USA) according to the instructions. Subsequently, 500 ng of total RNA was reverse-transcribed to first-strand cDNA by the PrimeScrip RT reagent Kit with gDNA Eraser (TaKaRa, Dalian, China) according to the manufacturer’s protocol, and the cDNA was diluted 10-fold for quantitative real-time PCR (qRT-PCR). qRT-PCR was performed using 2 μL of cDNA in a 20 μL reaction volume with SYBR^®^ Premix Ex Taq™ II (TaKaRa, Dalian, China) on a StepOnePlus Real-Time PCR System (ABI, USA), using the following PCR program: 95 °C for 3 min, followed by 40 cycles at 95 °C for 30 s and at 60 °C for 1 min 30 s. Melting curves were obtained to verify the amplification specificity through a stepwise heating of the amplicon from 60 to 95 °C. Primer pairs were designed by Primer Premier 5.0 (Table S4). The *GAPDH* gene was used as an internal control gene. Three independent biological replicates were performed, and the relative expression levels of the *DcaHsf* genes were calculated with the 2^−∆∆*Ct*^ method [53].

## 5. Conclusions

In this study, 17 *DcaHsf* genes were identified in the carnation genome for the first time. Comprehensive analyses of these genes, including phylogeny, genes structure, conserved motifs, and expression profiles in various tissues and under abiotic stresses were performed. Structural characteristics and comparisons with *A. thaliana*, *O. sativa*, and *P. trichocarpa* assisted in classifying these genes into three major classes (A, B, and C), with members of class A being the most abundant. The *DcaHsf* members were expressed in at least one tissue among root, stem, calyx, young leaf, mature leaf, stigma, ovary, and flower. In addition, *DcaHsfA1, A2a, A9a, B2a, B3a* revealed their putative involvement in the early flowering stages. The results of qRT-PCR revealed that all *DcaHsfs* responded to heat and drought, and many *DcaHsfs* were also regulated by cold, salt, and osmotic stress, as well as by the phytohormones ABA and SA. Our research suggests that *DcaHsf A2a*, *A2b* may be used as candidate genes for the breeding of heat-resistant carnation. Meanwhile, *DcaHsf-B2a, B3a* and *DcaHsfA5, B2b* could be considered as probable candidate genes for promoting early blooming and prolonging florescence in carnations.

## Figures and Tables

**Table 1:** Summary information of *Dianthus caryophyllus* heat shock transcription factors (DcaHsfs) in carnation. Notes: I.I., instability index; Stability, (U: unstable protein, S: stable protein); A.I., aliphatic index; n.c.r., total number of negatively charged residues (Asp + Glu); p.c.r., total number of positively charged residues (Arg + Lys); GRAVY, grand average of hydropathicity; pI, isoelectric point; MW, molecular weight.

Protein Name	Gene ID	Subfamily	Size	I.I.	Stability	A.I.	n.c.r. (%)	p.c.r.(%)	GRAVY	pI	MW (kDa)
DcaHsf-A1	*Dca57201.1*	A1	488	55.97	U	66.72	69	47	−0.690	4.74	54.49
DcaHsf-A2a	*Dca14360.1*	A2	380	59.24	U	83.55	59	45	−0.503	5.12	42.96
DcaHsf-A2b	*Dca52568.1*	A2	359	58.97	U	80.03	57	43	−0.548	5.05	40.55
DcaHsf-A3	*Dca41810.1*	A3	244	64.58	U	67.33	67	50	−0.566	4.98	51.75
DcaHsf-A4	*Dca23163.1*	A4	390	47.75	U	71.95	59	47	−0.859	5.7	44.99
DcaHsf-A5	*Dca19769.1*	A5	489	48.53	U	72.17	67	52	−0.745	5.45	54.52
DcaHsf-A7	*Dca4574.1*	A7	425	48.11	U	67.36	63	61	−0.771	6.72	48.91
DcaHsf-A9a	*Dca9629.1*	A9	401	47.92	U	69.73	67	46	−0.691	5	46.12
DcaHsf-A9b	*Dca41703.1*	A9	331	36.63	S	69.43	45	44	−0.805	6.23	38.21
DcaHsf-B1	*Dca60410.1*	B1	276	34.47	S	68.77	38	39	−0.804	7.61	31.0
DcaHsf-B2a	*Dca22545.1*	B2	337	54.72	U	61.64	35	29	−0.721	6	36.48
DcaHsf-B2b	*Dca48996.1*	B2	337	60.89	U	60.98	39	32	−0.709	6	36.48
DcaHsf-B2c	*Dca54105.1*	B2	318	61.35	U	68.57	33	32	−0.592	5.91	33.80
DcaHsf-B3a	*Dca44175.1*	B3	244	48.80	U	70.33	31	37	−0.763	8.88	28.38
DcaHsf-B3b	*Dca48010.1*	B3	457	48.40	U	68.96	31	37	−0.784	8.88	28.33
DcaHsf-B4	*Dca39623.1*	B4	287	54.07	U	72.30	30	32	−0.874	8.52	33.72
DcaHsf-C1 (incomplete)	*Dca24054.1*	C1	133	58.77	U	76.24	21	22	−0.881	8.01	15.89

**Table 2:** Analysis and distribution of conserved motifs in carnation DcaHsfs.

Motif	E-Value	Width	Best Possible Match
Motif1	2.70 × 10^−426^	41	VWDPAEFARDLLPRYFKHNNFSSFVRQLNTYGFRKVVPDRW
Motif2	5.20 × 10^−225^	29	PFLTKTYDMVDDPSTDDIVSWSEDGTSFV
Motif3	1.10 × 10^−187^	29	EFANEGFLRGQKHLLKNIKRRKTTTAHSQ
Motif4	4.20 × 10^−115^	41	GLEGENERLRRENEVLMSELVKLKQQQQNTFSLLQAMESRL
Motif5	4.80 × 10^−54^	34	QSTEWKQKQMMTFLAKAMQNPTFVQQLVQKKDER
Motif6	1.60 × 10^−22^	49	PPQQQPSTAAPTNSSDEQVISNSNSPPLAIPSVIMHRHHHHHHLYHNNN
Motif7	1.20 × 10^−19^	41	KSVKAIRVSMKRRLTSTLSAPNLNDVVEPELVRSMAVSSDN
Motif8	2.60 × 10^−16^	50	CGGGGGGSPMIFGVSIGGKRGREGGDDGGGEVVGGGEGLGATEVHDDHMH
Motif9	2.60 × 10^−13^	50	ATLDNGTDGDIKEQKVDDSMPPEIDTNVGDVSQTSWEELLWAEDEEGFRQ
Motif10	7.70 × 10^−10^	29	MRELSIKGLFDDHDDDDECGIIMRRKMTK
Motif11	1.50 × 10^−9^	13	PKPMEGLNEMNPP
Motif12	1.80 × 10^−5^	41	DSDGDDGNNKNRPKLFGVRLDLQDESERKRRKKLALDYTRT
Motif13	5.20 × 10^−8^	21	NNNNNNNVVITRKNNENEMNN
Motif14	1.40 × 10^−4^	29	PVENVVPESGNWGEDVEDLIEQLGFLGPM
Motif15	1.80 × 10^−4^	21	MTAVLVTVSDLVSSSTTSSSS
Motif16	2.10 × 10^−4^	29	MSPPPSPPAEEKPEKLTAVVVGGGGGETQ
Motif17	1.70 × 10^−3^	21	AAPSRVNDAVWTQLLTLPRGS
Motif18	2.90 × 10^−3^	10	GFRKVDPDKW
Motif19	1.30 × 10^−2^	23	TSTTCTCTPLSTESPQLGLQLSP
Motif20	1.00 × 10^−2^	19	YWYDFDGEDEVELEERVPC

**Table 3:** Functional domains of DcaHsfs.

Gene Name	DBD	HR-A/B	NLS	NES	RD	AHA
DcaHsf-A1	31–124	162–182/201–212	N.D.	(403) L	N.D.	N.D.
DcaHsf-A2a	40–154	183–201/222–233	(147–156) KTIKRRRNVT (258–267) AGMKRRLTST	N.D.	N.D.	(329–338) QTSWEELLWA
DcaHsf-A2b	40–133	162–180/201–212	(126–137) LLKTIKRRRNVT (237–246) AGMKRRLTST	(232–237) LDITHL	N.D.	(308–317) QTSWEELLWA
DcaHsf-A3	37–148	181–199/220–230	N.D.	(319) L	N.D.	N.D.
DcaHsf-A4	11–104	139–157/178–189	(204–213) HDRKRRFSRP	N.D.	N.D.	(325–334) DVFWEQFLTE
DcaHsf-A5	20–113	138–156/177–187	(206–217) LSAYNKKRRLPP	N.D.	ND	(438–447) DLFWEQFLTE
DcaHsf-A7	40–133	164–182/203–213	(126–140) LLKNIKRRKNPSQTF (237–246) LSKKRRRPIE	N.D.	ND	(322–331) DDFWEDLLNE
DcaHsf-A9a	87–180	202–220/241–251	(173–184) LLKSIKRKRHGS (275–285) RVSKKRRLAST	(204) LDQEALKVEI	ND	N.D.
DcaHsf-A9b	95–188	210–228/249–259	(181–192) LLKSIKRKRHGS (283–293) RVSKKRRLAST	(217–221) LKVEI	ND	N.D.
DcaHsf-B1	6–99	125–131	N.D.	(155–157) LEL	(220-226) KLFGVWL	N.D.
DcaHsf-B2a	32–125	151–169/190–200	N.D.	N.D.	ND	N.D.
DcaHsf-B2b	32–125	151–169/190–200	N.D.	N.D.	ND	N.D.
DcaHsf-B2c	26–119	145–153/172–178	N.D.	(197–202) KENMSL	(284–290) KLFGVSI	N.D.
DcaHsf-B3a	29–122	147–152	(5–30) SIKGLFDDHDDDDECGIIMRRKMTKP (178–187) NAMKRKCQEL (207–235) KNRPKLFGVRLDLQDESERKRRKKLALDY	(222–238) LDLQDESERKRRKKLAL	(216–222) KLFGVRL	N.D.
DcaHsf-B3b	29–122	147–152	(5–30) SIKGLFDDHDDDDECGIIMRRKMTKP (178–187) NAMKRKCQEL (207–235) KNRPKLFGVRLDLQDESERKRRKKLALDY	N.D.	(216–222) KLFGVRL	N.D.
DcaHsf-B4	12–105	131–149/163–166	(275–283) HSKKRLHLA	N.D.	(268–274) RLFGVPL	N.D.
DcaHsf-C1 (not full)	1–44	73–89/98–108	N.D.	(66) L	N.D.	N.D.

N.D., not detected.

## Supplementary Materials

Supplementary Materials can be found at https://www.mdpi.com/1422-0067/20/20/5233/s1.

## Abbreviations

ABA	Abscisic acid
ABRE	ABA-responsive element
APX	Ascorbate peroxidase
CA	Calyx
CAT	Catalase
DBD	DNA-binding domains
ERE	Ethylene-responsive element
F	Flower
FS	Flowering stages
HSE	Heat shock element
Hsf	Heat shock transcription factor
MEME	Multiple Em for Motif Elicitation
ML	Mature leaf
MW	Molecular weight
NES	Nuclear export signals
NLS	Nuclear localization signals
OV	Ovary
PEG	Polyethylene glycol
qRT-PCR	Quantitative real-time PCR
R	Root
RD	Repressor domains
ROS	Reactive oxygen species
S	Stem
SA	Salicylic acid
ST	Stigma
TFs	Transcription factors
YL	Young leaf
